# Telemental Health Collaborative Care Medication Management: Implementation and Outcomes

**DOI:** 10.1089/tmj.2021.0401

**Published:** 2022-07-04

**Authors:** Smita Das, Jane Wang, Shih-Yin Chen, Connie E. Chen

**Affiliations:** ^1^ Lyra Health, Burlingame, California, USA.; ^2^ Internal Medicine Residency Program, David Geffen School of Medicine, University of California, Los Angeles, Los Angeles, California, USA.

**Keywords:** telemedicine, telemental health, collaborative care, digital health

## Abstract

**Introduction:**

Access to quality mental health medication management (MM) in the United States is limited, even among those with employment-based health insurance. This implementation, feasibility, and outcome study sought to design and evaluate an evidence-based telemental health MM service using a collaborative care model (CoCM).

**Materials and Methods:**

CoCM MM was available to adult employees/dependents through their employer benefits, in addition to therapy. Outcomes included Patient Health Questionnaire-9 (PHQ-9) and the Generalized Anxiety Disorder-7 (GAD-7) collected at baseline and throughout participation. This analysis was not deemed to be human subjects research by the Western Institutional Review Board.

**Results:**

Over 17 months, 212 people enrolled and completed >2 assessments; the enrollees were 58.96% female with average age of 32.00 years (standard deviation [SD] = 7.38). In people with moderate to severe depression or anxiety, PHQ-9 and GAD-7 scores reduced by an average of 7.27 (SD = 4.80) and 6.71 (SD = 5.18) points after at least 12 ± 4 weeks in the program. At 24 ± 4 weeks, the PHQ-9 and GAD-7 reductions were on average 7.17 (SD = 5.00) and 6.03 (SD = 5.37), respectively. Approximately 65.88% of participants with either baseline depression or anxiety had a response on either the PHQ-9 or GAD-7 at 12 ± 4 weeks and 44.71% of participants experienced remission; at 24 ± 4 weeks, 56.41% had response and 41.03% experienced remission.

**Conclusions:**

An evidence-based CoCM telemedicine service within an employee behavioral health benefit is feasible and effective in reducing anxiety and depression symptoms when using measurement-based care. Widespread implementation of a benefit like this could expand access to evidence-based mental health MM.

## Introduction

The Coronavirus 2019 pandemic has intensified an already concerning mental health crisis in the United States. It has been suggested that collaborative care models (CoCMs) can help improve this crisis.^1^ While CoCMs have been studied, validated in randomized trials, and recommended for nearly 20 years, we still face a mental health shortage.^2^ Nearly one in five people has a mental health condition, but 60% of adults do not presently receive the mental health treatment they need.^3^ At the same time, 13.2% of American adults report past month antidepressant use, so they may be receiving some care, but likely from primary care, taking legacy medications, and perhaps some in CoCMs.^4–6^

While national guidelines recommend implementation of collaborative models to expand access to mental health care, there are many barriers to effective implementation, including provider dissatisfaction, difficulty tracking outcomes, lack of communication, lack of education, turnover, and insufficient staff time, especially when addressing mental health, in addition to other health concerns.^7–10^

Ideally, the collaborative model involves integration of evidence-based care and measurement-based care.^1,11–13^ With reference to evidence-based care, there are many guidelines developed to inform the treatment of depression, but even in algorithm dissemination studies, individual physician practice patterns continue to vary widely and adherence can be as low as 50%.^14–17^ One reason is that those guidelines can be burdensome. Over time, decisions in prescriptions may be guided more by clinical judgment and experience, rather than evidence-based guidelines, although a balance of these factors is important in medicine.^18,19^

In addition to poor provider adherence, structured algorithm studies have shown that patient nonadherence or dropout from psychiatric care is high; for depression algorithms, it ranged from 30% to 40%, which can reduce efficacy of CoCMs.^20^ Measurement-based care, another pillar of collaborative mental health care, is also pivotal to providing evidence-based care.^21^ However, many systems frame the implementation of measurement-based care as a burden or an imposition on the provider.^22^

Technology has great potential to help address the mental health care crisis and some of the gaps existing in CoCMs.^23^ Through telemedicine, digital consultations, measurement-based care outcomes, and evidence-based clinical support, more patients are able to access high-quality, evidence-based care for a larger proportion of their time in care. Patient concerns can be addressed more quickly due to the convenience of the interface. It is critical to integrate technology thoughtfully and balance it with the most important part of any care system, the therapeutic alliance.^24,25^

Prior studies have attempted to create telemedicine models of CoCMs for mental health.^26–28^ These models have shown to be effective in providing mental health care in the primary care setting, where a primary care provider may refer patients to an off-site psychiatrist for virtual mental health care. However, within these models, psychiatry-trained providers remain the main mental health providers interfacing with patients, rather than the primary care providers. This may not adequately relieve the burden of mental health provision within the United States, particularly given the imbalanced ratio of internists and family medicine providers to psychiatrists. Furthermore, these groups did not incorporate an avenue for constant direct access to their mental health providers to manage nuanced symptom changes.

To date, no literature or model to our knowledge has implemented a telemedicine-based collaborative care approach where (1) primary care physicians have constant direct access to a psychiatrist consultant with dedicated out-of-visit time, (2) evidence-based protocols, and measurement-based mental health-specific (rather than general primary) care, (3) patients can receive care at their desired location, and (4) patients possess direct access to their providers to react nimbly to nuanced changes in symptoms. Herein, we summarize the development, feasibility, and initial outcomes of a technology-enhanced CoCM service available to employees through their employer.

## Materials and Methods

### DEVELOPMENT OF CLINICAL SERVICE AND SETTING

Lyra Clinical Associates, a provider group that partners with Lyra Health, first offered their Medication Management (MM) service in November 2019. Lyra Health offers a behavioral health benefit to companies through which employees and dependents have access to video-based therapy, behavioral health coaching, and MM consultations; the existing programs incorporate rigorous quality assurance elements, including regular ongoing individual and team-based clinical consultation and clinical case reviews informed by routine symptom monitoring, elements that are central to CoCMs.

Both the therapy and coaching programs use digital materials (video lessons, exercises, and virtual handouts) that are based primarily in cognitive behavioral therapy with behavioral health coaches caring for clients who are clinically appropriate for a coach level provider. A portion of patients were enrolled in more than one treatment modality (coaching, therapy, and MM) concurrently. (MM to be described in detail below.)

Before offering MM, we conducted a review of the evidence-based guidelines for clinical assessment, measurement-based care, prescribing, shared decision making, and collaborative care. This clinical basis was translated into a formalized protocol for technology (for service delivery) and training of the physicians delivering care. Participants who sought help for mental health-related concerns went through an online onboarding process and answered questions about their symptoms, the impact of symptoms on their general functioning, and their interest in receiving care through video. Those who either had interests in controlled substances or were not open to seeing a provider through video were referred to in-person providers. Participants completed an up to 90-min consultation informed by an electronic intake form and scheduled follow-up visits as clinically necessary.

### STUDY DESIGN

This was a retrospective analysis using existing data from the MM program. All participants who had enrolled in the MM program completed standardized measures of depression and anxiety (Patient Health Questionnaire-9 [PHQ-9] and the Generalized Anxiety Disorder-7 [GAD-7]) at baseline and intermittently between visits.^29,30^ Participants had access to a minimum of 12 sessions depending on the benefit offered by the sponsoring company and length of care was not pre-specified. Sessions were considered a live video visit with a provider; digital content such as video lessons, exercises, assessments, and guides were provided between sessions. This analysis of deidentified data gathered from treatment offered by Lyra Health was not deemed to be human subjects research by the Western Institutional Review Board.

### PARTICIPANTS

Participants were individuals who started treatment in MM between November 1, 2019, and April 1, 2021. People at eligible companies were offered an MM consultation when they searched for care. Exclusion criteria were listed on the enrollment website and included needing prescriptions for controlled substances due to Ryan Haight Act limitations, being younger than 18 years, active suicidality/self-harm, and active homicidality. If in-person care was indicated, then the individual would be appropriately referred with a formal hand off to the care team. Furthermore, if patients participated in the consultation and were more appropriate for therapy, they could be referred to therapy instead of or in conjunction with the MM program.

**Fig. 1.**
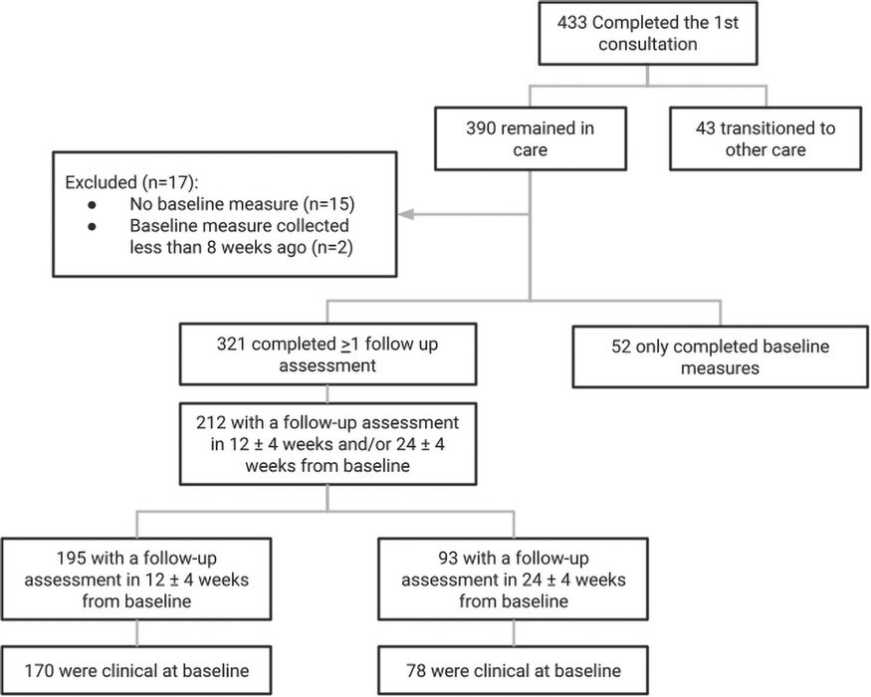
Participant flow: “Clinical” in the final steps of the flow indicates moderate to severe symptoms on measures.

The scores used for analyses were the assessments collected between 8 and 16 (12 ± 4) weeks after the baseline and the assessments collected between 20 and 28 (24 ± 4) weeks after the baseline; participants who were referred out from the program were not included in the analysis (*n* = 43) and people with baseline that was taken less than 8 weeks from the time of the data pull were also excluded (*n* = 2). For simplicity, henceforth, these time intervals will be referred to as 12 and 24 weeks. Therefore, of the 433 initial enrollees, 212 people who have a baseline measure and a follow-up assessment between 8 and 16 weeks and/or a follow-up assessment between 20 and 28 weeks after the baseline are included in the analysis, or 48.96% (*Fig. 1*).

### SELF-REPORTED MEASURES

Patients completed electronically secure online assessment questionnaires at baseline and between follow-up visits. Assessments consisted of the PHQ-9 and the GAD-7, well-validated measures of depression and anxiety.^29,30^ For depression, scores of 4 and below indicate no to minimal symptoms, 5 to 9 indicate mild symptoms, and above 10 are moderate and severe symptoms. For anxiety, the score of 4 and below on the GAD-7 indicate no to minimal symptoms as well; a clinical cutoff of 8 was used for the GAD-7 for moderate to severe symptoms, as research suggests that scores at or above 8 are highly likely to correspond to an anxiety disorder diagnosis.^31,32^

### TREATMENT

The MM program combined live video-based sessions with Board Certified Internal Medicine and Family Medicine physicians plus technology-based care education. These included digital video lessons (e.g., on understanding anxiety) and exercises/guides (such as self-assessing for goals), as well as assessments/check-ins (such as symptom reporting) that clients had access to use in between sessions; physicians were trained in assigning digital content to supplement clinical care. All treating physicians had access to a psychiatrist consultant for case and care review. All physicians were provided with continuing medical education before accepting clients focused on evidence-based practices and guidelines. Physicians had protected time for administrative duties, completion of thorough notes, review of patient data between visits, and care coordination with other providers.

In traditional CoCM models, a 30- to 60-min intake and shorter follow-up would address all health concerns, in addition to mental health; this model offered a deep dive into mental health with a 90-min intake and 30-min follow-ups. Another aspect novel to this model is continuous quality assurance, training, and consultation: sessions were recorded with participants' consent, and quality adherence was ensured through session review, weekly supervision video calls, and video consultation groups with other physicians.

### DIGITAL COMPONENTS OF MM

MM sessions were conducted on a secure, Health Insurance Portability and Accountability Act-compliant video platform. Following each session, physicians assigned information sheets about medications and assessments to be completed between sessions. They could also provide digital lesson videos, which consisted of animated videos and quizzes to test for comprehension and provide corrective feedback. The digital lessons utilized a storytelling approach where viewers followed the therapy journey of multiple characters presenting with symptoms of depression or anxiety. This approach has been used in other efficacious internet-delivered cognitive behavior therapy interventions, and it was found to have a normalizing effect for clients.^33^

### DATA ANALYSES

To estimate the impact of MM, we conducted paired two-tailed *t*-tests between baseline and last available assessment scores for each measure. We calculated Cohen's *d*_rm_, a conservative measure of effect size for within-subjects designs that controls for the correlation between measurements. We also summarized the response (50% reduction in PHQ-9 or GAD-7) scores at follow-up and remission among those who had measurable anxiety or depression at baseline and were subthreshold at follow-up (<5 on PHQ-9 or GAD-7).

## Results

Two hundred twelve participants completed the initial consultation, and had a follow-up PHQ-9 and GAD-7 assessment at least 8 weeks from their initial assessment. Participants' mean age was 32.00 years (standard deviation [SD] = 7.38); 58.96% (*n* = 125) of participants were women and 41.04% (*n* = 87) were men. Average number of visits was 5.74 (SD = 2.54). Around 58.02% of clients had at least two therapy sessions between the first and last assessments included in the analysis and 0% of clients had any coaching session in that time (*Table 1*).

**Table 1.** Participant Baseline and Care Characteristics

VARIABLE	PARTICIPANTS WITH >1 FOLLOW-UP ASSESSMENT AT LEAST 8 WEEKS AFTER BASELINE (*n* = 212); *n* (%) SHOWN UNLESS OTHERWISE NOTED
Gender	
Female	125 (58.96%)
Male	87 (41.04%)
Age group	
18–24	27 (12.74%)
25–34	127 (59.91%)
35–44	46 (21.70%)
45–54	9 (4.25%)
≥55	3 (1.42%)
Clinical presentation	
PHQ-9 < 10 (mild/minimal)	55 (25.94%)
PHQ-9 ≥ 10 (moderate/severe)	157 (74.06%)
GAD-7 < 8 (mild/minimal)	51 (24.06%)
GAD-7 ≥ 8 (moderate/severe)	161 (75.94%)
PHQ-9, mean (SD)	13.02 (5.37)
GAD-7, mean (SD)	11.73 (5.28)
% Suicidality	56 (26.42%)
Time in care, mean days (SD)	174.53 (98.82)
No. of visits, mean (SD)	5.74 (2.54)
≥2 Therapy sessions (through Lyra) during MM episode	123 (58.02%)
≥2 Coaching sessions (through Lyra) during MM episode	0 (0%)
Other medication visit (through Lyra) before MM	19 (8.96%)

GAD-7, generalized anxiety disorder-7; MM, medication management; PHQ-9, patient health questionnaire-9; SD, standard deviation.

### DEPRESSIVE SYMPTOMS

At pre-treatment, mean PHQ-9 score was 13.02 (SD = 5.37). Approximately 26.42% of participants reported some amount of suicidality on PHQ-9. Breakdown of depression severity according to PHQ-9 was 25.94% with mild/minimal depression (PHQ-9 < 10), 74.06% with moderate/severe depression. Results of paired *t-*tests revealed that for people with moderate-severe depression, PHQ-9 scores decreased from baseline to follow-up assessment. At 12 weeks, participants improved an average of 7.27 points (SD = 4.80), *t*(145) = 18.30, *p* < 0.001, Cohen's *d*_rm_ = −1.64; and at 24 weeks, scores improved an average 7.17 points (SD = 5.00), *t*(65) = 11.64, *p* < 0.001, Cohen's *d*_rm_ = −1.54, both suggesting a large effect of treatment on depression symptoms (*Table 2*). Of people with moderate-severe depression, 58.90% had a positive response at 12 weeks and 51.52% had a positive response at 24 weeks; 24.66% had remission at 12 weeks and 24.24% had remission at 24 weeks (*Table 3*).

**Table 2.** Baseline and Follow-Up Outcome Differences in Patient Health Questionnaire-9 and Generalized Anxiety Disorder-7 Scores in Individuals with Moderate to Severe Depression and/or Anxiety at Baseline with ≥8 Weeks of Outcomes

MEASURE	*N*	BASELINE OUTCOME MEAN (SD)	FOLLOW-UP OUTCOME MEAN (SD)	PAIRED DIFFERENCES MEAN (SD)	95% CONFIDENCE INTERVAL OF THE DIFFERENCE		*t*-VALUE (df)	*p*	COHEN'S *d*
					LOWER	UPPER			
12-Week outcomes									
PHQ-9	146	15.45 (3.74)	8.18 (4.95)	−7.27 (4.80)	−8.06	−6.49	18.30 (145)	<0.001	−1.64
GAD-7	149	14.13 (3.84)	7.42 (4.92)	−6.71 (5.18)	−7.55	−5.87	15.80 (148)	<0.001	−1.51
24-Week outcomes									
PHQ-9	66	15.80 (3.68)	8.64 (5.28)	−7.17 (5.00)	−8.40	−5.94	11.64 (65)	<0.001	−1.54
GAD-7	70	13.87 (3.77)	7.84 (4.99)	−6.03 (5.37)	−7.31	−4.75	9.39 (69)	<0.001	−1.35

**Table 3.** Response and Remission Rates

12-WEEK OUTCOMES				
*n* (%)	BASELINE DEPRESSION (*n* = 146) PHQ-9	BASELINE ANXIETY (*n* = 149) GAD-7	BASELINE DEPRESSION OR ANXIETY (*n* = 170)	BASELINE DEPRESSION AND ANXIETY (*n* = 125)
Response at 12 weeks				
50% improvement PHQ-9	86 (58.90%)	79 (53.02%)	96 (56.47%)	69 (55.20%)
50% improvement GAD-7	73 (50.00%)	77 (51.68%)	88 (51.76%)	62 (49.60%)
Response on either measure	96 (65.75%)	95 (63.76%)	112 (65.88%)	79 (63.20%)
Remission at 12 weeks				
PHQ-9 < 5	36 (24.66%)	43 (28.86%)	53 (31.18%)	26 (20.80%)
GAD-7 < 5	50 (34.25%)	44 (29.53%)	62 (36.47%)	32 (25.60%)
Remission on either measure	58 (39.73%)	58 (38.93%)	76 (44.71%)	40 (32.00%)
Response or remission on either at 12 weeks	98 (67.12%)	97 (65.10%)	116 (68.24%)	79 (63.20%)

24-WEEK OUTCOMES				
n (%)	BASELINE DEPRESSION (n = 66) PHQ-9	BASELINE ANXIETY (n = 70) GAD-7	BASELINE DEPRESSION OR ANXIETY (n = 78)	BASELINE DEPRESSION AND ANXIETY (n = 58)
Response at 24 weeks				
50% improvement PHQ-9	34 (51.52%)	28 (40.00%)	35 (44.87%)	27 (46.55%)
50% improvement GAD-7	29 (43.94%)	28 (40.00%)	32 (41.03%)	25 (43.10%)
Response on either measure	40 (60.61%)	37 (52.86%)	44 (56.41%)	33 (56.90%)
Remission at 24 weeks				
PHQ-9 < 5	16 (24.24%)	13 (18.57%)	17 (21.79%)	12 (20.69%)
GAD-7 < 5	27 (40.91%)	23 (32.86%)	29 (37.18%)	21 (36.21%)
Remission on either measure	29 (43.94%)	26 (37.14%)	32 (41.03%)	23 (39.66%)
Response or remission on either at 24 weeks	40 (60.61%)	37 (52.86%)	44 (56.41%)	33 (56.90%)

### ANXIETY SYMPTOMS

At pre-treatment, GAD-7 score was 11.73 (SD = 5.28). Breakdown of anxiety severity according to GAD-7 was 24.06% with mild/minimal anxiety (GAD-7 < 8), 75.94% with moderate/severe anxiety. Results of paired *t-*tests revealed that for people with moderate-severe anxiety, GAD-7 scores decreased. At 12 weeks, participants improved an average of 6.71 points (SD = 5.18), *t*(148) = 15.80, *p* < 0.001, Cohen's *d*_rm_ = −1.51; and at 24 weeks, participants showed an average improvement of 6.03 points (SD = 5.37), *t*(69) = 9.39, *p* < 0.001, Cohen's *d*_rm_ = −1.35, both suggesting a large effect of treatment on anxiety symptoms. Of people with moderate/severe anxiety, 51.68% had a positive response at 12 weeks and 40.00% had a positive response at 24 weeks; 29.53% had remission at 12 weeks and 32.86% had remission at 24 weeks.

### RESPONSE AND REMISSION

Of people with either moderate/severe depression or anxiety at baseline, 65.88% of patients demonstrated response either on PHQ-9 or GAD-7 at 12 weeks and 56.41% at 24 weeks; 44.71% demonstrated remission either on GAD-7 or PHQ-9 at 12 weeks and 41.03% at 24 weeks. Around 68.24% and 56.41% experienced response or remission on either scale at 12 and 24 weeks, respectively.

## Discussion

In a sample of 212 patients treated in a collaborative and technology-driven model, responses to mental health MM were appreciably higher than traditional modalities of care. Prior medication trials have reported response rates of 41–47%, and remission rates of 13–35% at 3 months of medication use for depression.^34,35^ For anxiety, prior literature has reported response rates of 63% and remission rates of 35% at 3 months of medication use.^36,37^ (Of note, the specific outcome measures in those studies may be different from the GAD-7 and PHQ-9.)

In research CoCM, response and remission from depression have been reported to be 35–46% and 20%, respectively.^7,38^ In more representative (not study selective) settings, the response and remission rates have been reported to be as low as 19% and 5%.^39^ In contrast, the results presented in this study achieve higher rates of response (>50%) and remission (>25%) in people with depression or anxiety at 12 weeks.

This is the first study examining outcomes in a collaborative and technology-driven mental health medication prescribing model that integrates digital tools, digital psychoeducational lessons, and measurement-based care and is focused only on mental health. As noted in the introduction, barriers to CoCMs include provider dissatisfaction, difficulty tracking outcomes, lack of communication, lack of education, turnover, and insufficient staff time, especially when addressing mental health, in addition to other health concerns.^7–10^

The model presented in this article differs from traditional CoCM models. While we do integrate elements of team, population, evidence, and measurement-based care, we focus the visit on mental health, rather than physical and mental health.^13^ The physicians in this model are Family Medicine and Internal Medicine physicians, so they have a broad understanding of medicine and complete a full medical history, but the visit focuses on the mental health concerns.

While in traditional models, a 30-min intake and 15-min follow-up would address all health concerns, in addition to mental health, this model offered a deep dive into mental health with a 90-min intake and 30-min follow-ups as clinically indicated. As a result, the physician can fully integrate guidelines, including a full review of psychiatric symptoms, history, substance use, risk assessment/planning, medical items, measurement-based care, shared decision making, and effective documentation.^17^

Time is uniquely protected for case consultation both individually and in a group with the psychiatrist, as well as for coordination of care with other providers (therapists, primary care physicians, etc.). (There was no routine case manager involved in the care.) The digital platform allowed for measurement-based care and seamless communication. All of these adjustments to traditional CoCM models may have improved outcomes. With only 1,907 psychiatry resident position offered in the 2021 National Resident Matching Program Main Residency Match versus 13,847 Internal or Family Medicine residency positions, we can meet the mental health shortage with creative solutions, combining psychiatrist leaders and mental health passionate primary care champions.^40^

Barriers to implementation center largely around adoption of services. Having members come to care continues to be a challenge; while we know that a greater percent of eligible members may benefit from a medication consultation by virtue of their severity, only 0.16% sought a consultation. We have instituted several efforts to increase member and provider (therapist) awareness to increase adoption. On the physician side, consulting with physicians in real time (during a visit) while we are at different time zones can be challenging, but we use messaging systems to aid; we also have established individual and group consultation times that are protected for case review.

Clinically, the other challenge has been with individuals who are interested in controlled substances, which we do not provide in the platform due to the Ryan Haight Act; those individuals are referred to our in-person network of providers. Feedback from members has been positive with the majority rating their visit as a 5 on a 5-point scale, and within our physicians' group, we have had 50% of the physicians from these data transition to full time from part time.

Aside from inherently being an uncontrolled data set, which limits the interpretation of the findings, another major limitation of the analysis is the inconsistency of follow-up periods. Among those with follow-up assessments, the timing of the end point is variable. To be conservative, we assumed an individual's last assessment was the endpoint, even if they remained in care for far longer. That endpoint may be well before the last session or up to 5 weeks after the last session. Therefore, comments on outcomes are reliant upon this assumption of timing. To add, if an individual's scores vacillated in treatment, that is not captured in this analysis.

It is also notable that improvement generally dropped between 3 and 6 months, possibly due to either worsening outcomes or response bias, where patients who are improved may be less likely to complete outcome assessments. Another limitation is about the chronicity of care. Some people seeking MM had plateaued with therapy, whereas others did not engage with therapy ever or engaged later. Changes in scores do not account for what part is attributable to MM only. Given that 58.02% are concurrently in therapy during their episode of care with MM, it is reasonable to assume that some of the gains in the outcomes were related to therapy, or that there was a synergistic effect from concurrent pharmacotherapy and therapy.^41^

The therapy service availability is a positive aspect of the model and another distinguishing feature of this analysis is that we have access to detailed data about therapy usage, which may not otherwise be available in the community. Future studies could compare similar cohorts in MM, therapy, and both to comment on attributable gains.

This study focuses on depression and anxiety outcomes. Other common diagnoses among those who had measurable anxiety or depression at baselines were bipolar (4.52%) and attention deficit hyperactivity disorder (13.87%). By limiting outcomes to PHQ-9 and GAD-7, we do not account for variability in outcomes for diagnosis-specific conditions, and instead offer a simple model.

In terms of population, the included sample may be unique. Unlike the preponderance of care models, MM participants have easy access to therapy in all cases (>50% of client searching for therapy in the care platform will be offered as appointment in the next 2 days with an average wait time of 6 days between booking and the first completed therapy appointment), so they may elect to complete a medication consultation solely due to personal preference. Some may be in therapy or may enter therapy. We noted that 13% of American adults take antidepressants, but 60% of Americans with mental health issues do not get the treatment that they need^3,4^; the choice of utilizing a psychiatric medication in the community may be in the absence of other resources and comes from a primary care physician without protected time or support.

Therefore our results may be impacted in one of two ways. First, included clients may self-select as more severe and want to access medications, while in usual care settings, medications may be the first point of access without therapy. On the other hand, these clients may have more robust outcomes because they have access to therapy resources.

Future research would account for covariables, which may affect outcomes such as therapy concurrency, choice of medication, diagnosis, completion of digital psychoeducation materials, impact of direct messaging with providers, and frequency of visits. A prospective randomized design would shed more light on the efficacy of this model. If limited to a cohort design, it would be useful to summarize data of a larger group over a longer period of time. Of note, that while 269,794 people were eligible for care in our model, only 433 (0.16%) sought a medication consultation. Since this service is efficacious and convenient, dissemination through awareness campaigns may help people connect with mental health services sooner.

## Conclusions

To summarize, developing a technology-enhanced CoCM service available to employees/dependents through their employer was feasible and demonstrated favorable outcomes on depression and anxiety. This type of digital clinic with access to therapy and coaching can improve access and reach of mental health. This model allows for better implementation of evidence-based care, designated time for collaboration, and preservation of the therapeutic relationship.
